# Determinants of Low Birth Weight among Deliveries at a Referral Hospital in Northern Ethiopia

**DOI:** 10.1155/2018/8169615

**Published:** 2018-04-23

**Authors:** Lema Desalegn Hailu, Deresse Legesse Kebede

**Affiliations:** ^1^School of Nursing and Midwifery, College of Health Sciences and Medicine, Wolaita Sodo University, Sodo, Ethiopia; ^2^School of Public Health, College of Medicine and Health Sciences, Hawassa University, Hawassa, Ethiopia

## Abstract

**Background:**

Low birth weight is the leading cause of infant and child mortality and contributes to several poor health outcomes. Proper knowledge of risk factors of low birth weight is important for identifying those mothers at risk and thereby for planning and taking appropriate actions. This study investigates factors predicting occurrence of low birth weight among deliveries at Debreberhan Referral Hospital.

**Methods:**

Facility-based unmatched case-control study was conducted among deliveries that took place at Debreberhan Referral Hospital. Birth records and mothers' ANC files were reviewed from April to June 2016. The study participants were selected by consecutive sampling technique. Data analysis was performed by SPSS version 20. Binary logistic regression analysis was performed to identify predictors of low birth weight.

**Result:**

A total of 147 birth records of babies with low birth weight (cases) and 294 birth records of babies with normal birth weight (controls) were reviewed. The birth weight of low birth weight babies (cases) ranged from 1000 grams to 2400 grams with median (±IQR) of 2200 grams (±300 grams), whereas it ranged from 2500 grams to 4500 grams with median (±IQR) of 3100 grams (±525 grams) among controls. Preterm birth (AOR = 5.32; CI = 2.959–9.567), history of any physical trauma experienced during pregnancy (AOR = 13.714; CI = 2.382–78.941), and history of any pregnancy complication (AOR = 2.708; CI = 1.634–4.487) were predictors of low birth weight. On the other hand, cesarean delivery (AOR = 0.415; CI = 0.183–0.941) and instrumental (AOR = 0.574; CI = 0.333–0.987) modes of delivery as well as maternal history of chronic diabetes (AOR = 0.275; CI = 0.090–0.836) had preventive effect of low birth weight.

**Conclusion:**

Preterm birth, history of experiencing any physical trauma during pregnancy, and history of any pregnancy complication were predictors of low birth weight, whereas cesarean and instrumental delivery had positive effect to preventing low birth weight.

## 1. Background

Birth weight or size at birth is an important indicator of the child's vulnerability to the risk of childhood illnesses and to predict the child's future health, development, and the chances of survival [1]. Low birth weight (LBW) is defined by the World Health Organization (WHO) as weight at birth of less than 2,500 grams. This is based on epidemiological observations that infants weighing less than 2,500 grams are at higher risk of neonatal mortality when compared with heavier babies [2]. Low birth weight is considered as the single most important predictor of infant mortality, especially of deaths within the first months of life [3]. Globally, 60–80% of neonatal deaths occur among low birth weight infants [1]. In developing countries, a birth weight below 2,500 grams is the leading cause of infant and child mortality and contributes to several poor health outcomes [2]. It is associated with poor neurological and cognitive development, childhood morbidity, growth impairment, a range of poor health outcomes, and chronic diseases later in life. It is a cause of both short-term and long-term consequences leading to adverse social and economic impacts [4, 5].

Globally, more than 20 million infants, representing 15.5% of all births, are born with low birth weight; 95.6% of them lived in developing countries, accounting for 17% of all births in developing countries [6, 7]. According to a hospital-based study among institutional deliveries in Iran, the prevalence of low birth weight was 40%, and gestational age less than 37 weeks, maternal age less than 20 years, irregular antenatal checkup, mother's height less than 150 cm, mother's weight less than 50 kg, hemoglobin less than 10 gm/dl, severe physical work, and tobacco chewing were significant determinants of low birth weight [8]. An epidemiological survey from China showed that low birth weight was associated with maternal age of less than 20 years, low level of maternal education, previous histories of adverse pregnancies, and pregnancy comorbidities and complications, such as hypertensive disorders during pregnancy, anemia, oligohydramnios, premature rupture of membranes, and gestational diabetes [9]. In a study from Lombok, Indonesia, determinants of LBW included infant's sex, woman's education, season at birth, mothers' residence, household wealth, maternal mid-upper arm circumference (MUAC), maternal height, birth order, and pregnancy interval [1]. According to the World Health Organization (WHO), factors contributing to LBW in developing countries include inadequate weight gain during pregnancy, low prepregnancy weight, short stature, malaria and other infectious diseases, hard physical work during pregnancy, social factors such as lower status of women, malnutrition, and lack of antenatal care [10]. In Ethiopia, the prevalence of LBW is as high as more than 10% according to reports from different regions of the country. A recent study reported from Tigray region, northern Ethiopia, showed that 10.5% of live births in the region were underweight [4]. According to a study conducted in Gondar University Hospital, northern Ethiopia, 11.2% of deliveries that were performed in the hospital were LBW [11], and in another hospital-based study from Tigray region, northern Ethiopia, the prevalence of low birth weight was 14.6% [7]. According to the national Ethiopian Demography and Health Survey 2011 (EDHS 2011), among babies who had reported birth weight in the country, 11% (17% in rural and 9% in urban) were born with low birth weight (weighed less than 2.5 kilograms), with variations among regions. Apart from regional variations, low birth weight was more common among children of the youngest mothers (age less than 20 years) and older mothers (age 35–49 years). Furthermore, first-order births, children of mothers with no education, and children born to mothers in the lowest wealth quintile were the most likely to be reported as very small [12]. On the other hand, factors like lack of antenatal follow-up, preterm delivery, chronic medical illness, lack of formal education, young age of the mother, and so forth were reported to be associated with low birth weight from different studies [4, 7, 13].

Having proper knowledge of risk factors for low birth weight is important to identification and giving appropriate attention to those mothers at risk. Despite few studies available to show factors associated with low birth weight across the country, it is not investigated in the study area. Therefore, this study investigates factors related to low birth weight particularly among deliveries at Debreberhan Referral Hospital. Findings from this study add to the current knowledge of risk factors of low birth weight particularly regarding mode of delivery, maternal chronic diabetes, pregnancy complication, and physical trauma during pregnancy which has not been well explained by other studies. This will have great relevance in identifying mothers and children at risk, designing appropriate measures, and undertaking timely interventions.

## 2. Methods and Materials

### 2.1. Study Design, Setting, and Population

Facility-based unmatched case-control study was conducted from April to June 2016 among deliveries that were performed at Debreberhan Referral Hospital. The hospital is found in Debreberhan town, located 130 kilometers to the North East of Addis Ababa. It is one of the most popular health facilities in the town rendering a range of medical, maternal, and child health services including delivery services, and various inpatient and outpatient healthcare services for a catchment population of over 95 thousand coming from the town as well as surrounding rural areas. Deliveries that take place at Debreberhan hospital were the source population. A case (low birth weight) was defined as a live birth baby born with birth weight of <2500 grams; and a control was defined as a live birth baby born with birth weight of ≥2500 grams.

### 2.2. Sample Size and Sampling Technique

Assuming odds ratio of 2 desired to detect, 95% confidence level, 80% power, and control to case ratio of 2, the sample size was calculated by Open Epi version 2.3 using the formula for unmatched case control and found to be 441, 147 cases and 294 controls. Birth records were selected by consecutive sampling technique. For each low birth weight (birth weight < 2500 grams), the next two delivery cases with birth weight ≥ 2500 grams were considered as controls.

### 2.3. Data Collection and Analysis

Data were collected by review of birth records and mothers' ANC files. Relevant data were extracted from the birth records as well as corresponding mothers' ANC files using a structured checklist. Sociodemographic as well as maternal and obstetric variables were extracted from birth records, while gestational age of the fetus and data about history of pregnancy complications were obtained from the mothers' ANC files. Data processing and analysis were performed by using Statistical Package for Social Sciences (SPSS) version 20. Having the average age and the relative proportion of mothers in each age category under consideration as well as other literatures for comparison purpose, age of the mothers was categorized into three as 15–24 years, 25–34 years, and 35 years or above. Birth weight was categorized into two as low birth weight (birth weight < 2500 grams), considered as cases, and normal birth weight (birth weight ≥ 2500 grams), considered as controls or the reference birth weight. Multicollinearity diagnostics was performed to check for collinearity among independent variables and evaluated that each of the independent variables in the multiple analysis had tolerance value >0.1 against every other independent variable. Multiple binary logistic regression analysis was performed to identify independent predictors of low birth weight. Adjusted odds ratio (AOR) with 95% confidence interval (CI) and *p* value ≤ 0.05 were used to claim statistical significance.

## 3. Result

### 3.1. Maternal Characteristics

A total of 147 birth records of babies with low birth weight (cases) and 294 birth records of babies with normal birth weight (controls) were reviewed in this study. The age of mothers ranged from 15 to 42 years with mean (±SD) of 25.37 (±4.99) years. Overall, majority of the mothers were in the age group of 15–24 years, followed by 25–34 years, accounting for 203 (46.0%) and 202 (45.8%), respectively. The median (±IQR) age of mothers was 24 (±7) years among cases, while it was 25 (±6) among controls. Among cases, 77 (52.4%) of mothers were in the age group of 15–24 years, while 146 (49.7%) of maternal age among controls were found in the age group of 25–34 years. The proportion of mothers aged 35 years and above were relatively small both in cases and in controls: 14 (9.5%) and 22 (7.5%), respectively. Maternal gravidity ranged from 1 to 6 with primigravida accounting for the larger proportion of mothers both in cases and in controls: 86 (58.5%) and 156 (53.1%), respectively. Maternal parity ranged from 0 to 5 with primipara accounting for the larger proportion of mothers both in cases and in controls: 90 (61.2%) and 172 (58.5%), respectively. ANC follow-up status of mothers was almost similar among cases and controls. Among cases, 54 (36.7%) of mothers had ANC visit, while the remaining 93 (63.3%) had no ANC visit. Among controls, 107 (36.4%) of mothers had ANC visit, while 187 (63.6%) had no ANC visit. The hemoglobin level of mothers ranged from 6.9 g/dl to 19.3 g/dl among cases with mean (±SD) of 13.6 g/dl (±1.93 g/dl), whereas in controls it ranged from 6.0 g/dl to 41.0 g/dl with mean (±SD) of 13.8 g/dl (±2.58 g/dl). The proportion of mothers who ever experienced any physical trauma during pregnancy was relatively higher among cases. Among cases, 5 (3.4%) of mothers had ever experienced any physical trauma during pregnancy, while the proportion was only 2 (0.7%) among controls. Among cases, 76 (51.7%) of mothers experienced any one or more signs of pregnancy complications (any one or more of bleeding, gush, headache, blurred vision, fever, and severe abdominal pain) during pregnancy, whereas among controls, 106 (36.1%) of mothers had any sign of pregnancy complications during pregnancy (Table 1).

### 3.2. Newborn Characteristics

The birth weight of babies ranged from 1000 grams to 2400 grams among cases with median (±IQR) of 2200 grams (±300 grams), whereas in controls it ranged from 2500 grams to 4500 grams with median (±IQR) of 3100 grams (±525 grams). Male age accounted for the larger proportion of cases, 78 (53.1%), while female age accounted for the larger proportion of controls, 158 (53.7%). Regarding gestational age at birth, 82 (55.8%) of cases were delivered at their full term, while 47 (32.0%) were delivered preterm or premature. Among controls, 231 (78.6%) of deliveries were full term, while 25 (8.5%) were preterm or premature. With regard to mode of delivery, the proportion of spontaneous vaginal delivery (SVD) was higher among cases as compared to controls. Among cases, 106 (72.1%) of the mode of delivery was SVD, whereas SVD accounted for 172 (58.5%) of the mode of delivery among controls. In contrast, the proportion of cesarean section (CS) and other instrumental deliveries were lower among cases as compared to controls (Table 1).

### 3.3. Bivariate Analyses

From binary logistic regression analyses of each variable with low birth weight, maternal age, gestational age at birth, mode of delivery, and experience of any sign of pregnancy complications were found to have significant association with low birth weight. Moreover, maternal experience of any physical trauma during pregnancy had remarkable effect on low birth weight. Independent samples *t*-test showed that there is no significant difference in the mean hemoglobin level of mothers between cases and controls (*p* = 0.349). Both maternal gravidity and parity in its continuous scale as birth order as well as the categorized forms had no significant association with low birth weight (Table 2).

### 3.4. Multivariable Analyses

From the multiple binary logistic regression analyses, gestational age at birth (preterm), mode of delivery, maternal history of chronic diabetes mellitus, experience of any physical trauma during pregnancy, and experience of any sign of pregnancy complications (any one or more of bleeding, gush, headache, blurred vision, fever, and severe abdominal pain) were found to be independent predictors of low birth weight. Babies who were born preterm (premature babies) were five times more likely to have low birth weight as compared to those born at their full term (AOR = 5.321; CI = 2.959–9.567). With regard to mode of delivery, babies born via cesarean delivery were 58% less likely to have low birth weight (AOR = 0.415; CI = 0.183–0.941) and babies born via instrumental delivery (vacuum delivery, forceps delivery, etc.) were 43% less likely to have low birth weight (AOR = 0.574; CI = 0.333–0.987) when compared to babies born via spontaneous vaginal delivery. On the other hand, babies born to mothers who had history of any physical trauma experienced during pregnancy were about fourteen times more likely to have low birth weight as compared to babies born to mothers who did not have such history (AOR = 13.714; CI = 2.382–78.941). And similarly, babies born to mothers who had history of any sign of pregnancy complications (any one or more of bleeding, gush, headache, blurred vision, fever, and severe abdominal pain) were about three times more likely to have low birth weight as compared to babies born to mothers who did not have such history (AOR = 2.708; CI = 1.634–4.487). Maternal history of chronic diabetes was found to have negative relationship with low birth weight. In this regard, babies born to mothers having history of chronic diabetes mellitus were 72% less likely to have low birth weight as compared to those born to mothers with no history of chronic diabetes mellitus (AOR = 0.275; CI = 0.090–0.836). The variables such as sex of the newborn, maternal age category, maternal parity (birth order), ANC visit, and maternal hemoglobin level had no significant association with low birth weight (Table 3).

## 4. Discussion

This study investigated some maternal and pregnancy related factors associated with low birth weight. Preterm birth was found to be a risk factor for low birth weight; babies born preterm were more likely to be of low birth weight when compared to their full term counterparts. This is supported by a study from Ardabil, Iran, in which preterm birth was reported to be a risk factor of low birth weight [14] and a study conducted in Kuala Lumpur, Malaysia, in which premature delivery was among predictors of low birth weight [5]. It is also in line with report from a study in Gondar University Hospital, northwest Ethiopia, in which preterm births were about six times more likely to be of low birth weight when compared to full term ones [11], and also supported by reports from hospital-based studies conducted in Tigray region, northern Ethiopia, where premature delivery (gestational age less than 37 weeks) was one of the important predictors of low birth weight [4, 7]. It is clear that babies born premature before completing their term due to any gynecological, medical, or other causes are not completing their normal physical development in the womb and at higher risk to have low weight at birth. In this regard, it could be important that any gynecological, medical, or other condition that could be possible cause of premature delivery should be timely recognized and properly managed during pregnancy.

In the current study, mode of delivery was found to be associated with low birth weight. It was observed that babies born via cesarean section as well as instrumental deliveries (vacuum delivery, forceps delivery, etc.) were less likely to be of low birth weight when compared to those born via spontaneous vaginal delivery. This means that low birth weight is less common among cesarean as well as instrumental deliveries while it is more common among spontaneous vaginal deliveries. It could be due to the natural mechanism that cesarean and instrumental procedures are more likely to be indicated for larger sized babies (the rates of cesarean and instrumental deliveries are lower for low birth weight babies), while relatively smaller sized babies with low birth weight are likely to be born spontaneously. Apart from this, the natural procedure in the case of spontaneous vaginal delivery could have impact on the birth weight of the baby. That is, series of physical compressions due to the labor process and the procedure during spontaneous vaginal delivery could have been resulting in significant weight loss to the baby during delivery. Although cesarean section and minor instrumental procedures like vacuum or forceps deliveries are applied for different reasons, they would have positive effect in maintaining birth weight of the baby. Those babies born via cesarean section and other instrumental procedures are prevented from potential compressions at birth and hence attributable weight loss during delivery, yet the association with mode of delivery needs further investigation.

It was found in the current study that occurrence of any sign of pregnancy complications (any one or more of bleeding, gush, headache, blurred vision, fever, and severe abdominal pain) was a risk factor for low birth weight. This is consistent with the finding from a study conducted in Ardabil, Iran, in which bleeding or spotting during pregnancy was reported to be a risk factor for low birth weight [14]. It was also supported by an epidemiological survey conducted in China [9], as well as a hospital-based study from Bale zone, southeast Ethiopia [13]. The signs and symptoms of pregnancy complications are indicative of some kind of disorder during pregnancy which could have negative impact on the birth weight of the baby. It is therefore recommended that pregnant women should be aware of danger signs during pregnancy and possible cause of such complications during pregnancy should be timely recognized and properly managed during pregnancy. To this end, regular antenatal follow-up by pregnant women might help for early detection and appropriate management of such disorders during pregnancy.

It was observed that babies born to mothers who had experienced any physical trauma during pregnancy were more likely to be born with low birth weight. The physical trauma could have directly resulted from an accidental injury which occurred during pregnancy or it could have resulted from a forceful hard work performed indoor or outdoor during pregnancy. According to a study conducted at a tertiary care hospital in Uttar Pradesh, severe physical work was one of the significant determinants of low birth weight [8]. In line, WHO reported that hard physical work during pregnancy is one of the factors contributing to occurrence of low birth weight in developing countries [11]. Any physical trauma encountered during pregnancy might have been accompanied by internal bleeding or soft tissue injury to the mother or the conception in the womb and could have significant impact on the birth weight of the outcome. As any physical trauma during pregnancy might have such negative impact on the birth weight of the outcome, avoiding hard work and preventing physical trauma are important during pregnancy.

According to this study, babies born to mothers having history of chronic diabetes mellitus were less likely to be born with low birth weight when compared to those born to mothers with no history of chronic diabetes mellitus. This could be explained by the medical effect that such mothers are medically likely to give birth to “giant babies” or the observed association could be just incidental. However, it is in contrary to the finding from a hospital-based study in Tigray, northern Ethiopia, which reported that presence of any chronic medical illness increased the risk of low birth weight [7]. The discrepancy could be related to the specific type of disease observed as evidenced from studies in northern Tanzania [15] as well as Gondar, Ethiopia [11], in which mother's chronic hypertension was observed to be associated with low birth weight or it could be related to variations in clinical stages of the diseases.

Generally, this study has identified risk factors of low birth weight particularly those pertaining to the study area and most of which has not been identified by other previous studies. Despite employing case-control design which we consider as the strength of this study, it is yet not without limitation. As variables related to maternal nutrition and biomedical or anthropometric variables of the mother such as height, weight, and body mass index might have effect on the birth weight of the pregnancy outcome, lack of these variables from secondary data is limitation of the study.

## 5. Conclusion

Preterm birth, history of experiencing any physical trauma during pregnancy, and history of any pregnancy complication were predictors of low birth weight, whereas cesarean and instrumental modes of delivery had preventive effect of low birth weight. Prompt identification of causes and prevention of premature delivery, proper knowledge of signs and symptoms of pregnancy complications, and preventing any physical trauma or its potential causes are recommended during pregnancy to prevent low birth weight. Identification of high risk mothers and early detection and management of the risk factors would reduce incidence of low birth weight and related short-term and long-term consequences.

## Figures and Tables

**Table 1:** Frequency distribution of maternal and newborn characteristics among cases and controls, Debreberhan Referral Hospital, northern Ethiopia, June 2016.

Variable	Cases (*n* = 147)		Controls (*n* = 294)	
	Frequency	%	Frequency	%
Sex of newborn				
Female	69	46.9%	158	53.7%
Male	78	53.1%	136	46.3%
Mothers' age category				
15–24	77	52.4%	126	42.9%
25–34	56	38.1%	146	49.7%
35–42	14	9.5%	22	7.5%
Mother's gravidity				
Primigravida	86	58.5%	156	53.1%
Multigravida	61	41.5%	138	46.9%
Mother's parity				
Primipara	90	61.2%	172	58.5%
Multipara	57	38.8%	122	41.5%
Mother's history of abortion				
No	137	93.2%	274	93.2%
Yes	10	6.8%	20	6.8%
Gestational age at birth				
Full-term	82	55.8%	231	78.6%
Preterm	47	32.0%	25	8.5%
Postterm	4	2.7%	11	3.7%
Unknown	14	9.5%	27	9.2%
Mode of delivery				
SVD	106	72.1%	172	58.5%
Instrumental	30	20.4%	86	29.3%
CS	11	7.5%	36	12.2%
Mother's ANC visit				
No	93	63.3%	187	63.6%
Yes	54	36.7%	107	36.4%
Iron supplementation during pregnancy				
No	129	87.8%	251	85.4%
Yes	18	12.2%	43	14.6%
Mother's history of chronic hypertension				
No	118	80.3%	239	81.3%
Yes	29	19.7%	55	18.7%
Pregnancy-induced hypertension (PIH)				
No	131	89.1%	276	93.9%
Yes	16	10.9%	18	6.1%
Mother's history of anemia				
No	117	79.6%	241	82.0%
Yes	30	20.4%	53	18.0%
Mother's history of chronic DM				
No	126	85.7%	238	81.0%
Yes	21	14.3%	56	19.0%
Experience of physical trauma during pregnancy				
No	142	96.6%	292	99.3%
Yes	5	3.4%	2	0.7%
Any sign of pregnancy complications				
No	71	48.3%	188	63.9%
Yes	76	51.7%	106	36.1%

**Table 2:** Bivariate analyses of different variables with low birth weight, Debreberhan Referral Hospital, northern Ethiopia, June 2016.

Variable	Cases (*n* = 147)	Controls (*n* = 294)	COR (95% CI)	*p* value
	Frequency (%)	Frequency (%)		
Sex of newborn				
Female	69 (46.9%)	158 (53.7%)	1.000	
Male	78 (53.1%)	136 (46.3%)	1.313 (0.883, 1.953)	0.178
Mothers' age category				
15–24	77 (52.4%)	126 (42.9%)	1.595 (1.048, 2.422)	0.029
25–34	56 (38.1%)	146 (49.7%)	1.000	
35–42	14 (9.5%)	22 (7.5%)	1.659 (0.794, 3.469)	0.178
Mother's gravidity		*β* = −0.039	0.962 (0.827, 1.118)	0.614
Gravidity in category				
Primigravida	86 (58.5%)	156 (53.1%)	1.000	
Multigravida	61 (41.5%)	138 (46.9%)	0.802 (0.537, 1.196)	0.279
Mother's parity		*β* = −0.035	0.965 (0.826, 1.128)	0.657
Parity in category				
Primipara	90 (61.2%)	172 (58.5%)	1.000	
Multipara	57 (38.8%)	122 (41.5%)	0.893 (0.596, 1.339)	0.583
History of abortion				
No	137 (93.2%)	274 (93.2%)	1.000	
Yes	10 (6.8%)	20 (6.8%)	1.000 (0.456, 2.195)	1.000
Gestational age at birth				
Term	82 (55.8%)	231 (78.6%)	1.000	
Preterm	47 (32.0%)	25 (8.5%)	5.296 (3.066, 9.149)	0.000
Postterm	4 (2.7%)	11 (3.7%)	1.024 (0.317, 3.306)	0.968
Unknown	14 (9.5%)	27 (9.2%)	1.461 (0.731, 2.921)	0.284
Mode of delivery				
SVD	106 (72.1%)	172 (58.5%)	1.000	
Instrumental	30 (20.4%)	86 (29.3%)	0.566 (0.350, 0.916)	0.020
CS	11 (7.5%)	36 (12.2%)	0.496 (0.242, 1.016)	0.055
ANC visit				
No	93 (63.3%)	187 (63.6%)	1.000	
Yes	54 (36.7%)	107 (36.4%)	1.015 (0.673, 1.531)	0.944
Iron supplementation				
No	129 (87.8%)	251 (85.4%)	1.000	
Yes	18 (12.2%)	43 (14.6%)	0.814 (0.452, 1.469)	0.495
Chronic hypertension				
No	118 (80.3%)	239 (81.3%)	1.000	
Yes	29 (19.7%)	55 (18.7%)	1.068 (0.647, 1.762)	0.797
PIH				
No	131 (89.1%)	276 (93.9%)	1.000	
Yes	16 (10.9%)	18 (6.1%)	1.873 (0.926, 3.789)	0.081
History of anemia				
No	117 (79.6%)	241 (82.0%)	1.000	
Yes	30 (20.4%)	53 (18.0%)	1.166 (0.708, 1.921)	0.547
Chronic DM				
No	126 (85.7%)	238 (81.0%)	1.000	
Yes	21 (14.3%)	56 (19.0%)	0.708 (0.410, 1.223)	0.216
Trauma during preg.				
No	142 (96.6%)	292 (99.3%)	1.000	
Yes	5 (3.4%)	2 (0.7%)	5.141 (0.985, 26.823)	0.052
Any sign preg. comp.				
No	71 (48.3%)	188 (63.9%)	1.000	
Yes	76 (51.7%)	106 (36.1%)	1.898 (1.270, 2.837)	0.002

**Table 3:** Multiple logistic regression analyses of different variables with low birth weight, Debreberhan Referral Hospital, northern Ethiopia, June 2016.

Variable	AOR (95% CI)	*p* value
Sex of newborn		
Female	1.000	
Male	1.246 (0.795, 1.954)	0.337
Mothers' age category		
15–24	1.454 (0.897, 2.357)	0.128
25–34	1.000	
35–42	1.662 (0.692, 3.990)	0.256
Mother's parity	0.961 (0.790, 1.169)	0.690
History of abortion		
No	1.000	
Yes	0.974 (0.391, 2.425)	0.955
Gestational age at birth		
Term	1.000	
Preterm	5.321 (2.959, 9.567)	0.000^*∗*^
Postterm	1.418 (0.413, 4.866)	0.579
Unknown	1.565 (0.735, 3.335)	0.246
Mode of delivery		
SVD	1.000	
Assisted	0.574 (0.333, 0.987)	0.045^*∗*^
CS	0.415 (0.183, 0.941)	0.035^*∗*^
ANC visit		
No	1.000	
Yes	0.864 (0.522, 1.430)	0.570
Mother's hemoglobin level (g/dl)	0.955 (0.863, 1.058)	0.378
Iron supplementation		
No	1.000	
Yes	0.607 (0.285, 1.290)	0.194
Chronic hypertension		
No	1.000	
Yes	1.226 (0.445, 3.380)	0.694
PIH		
No	1.000	
Yes	1.473 (0.625, 3.470)	0.376
History of anemia		
No	1.000	
Yes	2.172 (0.776, 6.074)	0.139
Chronic DM		
No	1.000	
Yes	0.275 (0.090, 0.836)	0.023^*∗*^
Trauma during pregnancy		
No	1.000	
Yes	13.714 (2.382, 78.941)	0.003^*∗*^
Any sign of preg. complications		
No	1.000	
Yes	2.708 (1.634, 4.487)	0.000^*∗*^

^*∗*^Statistically significant with *p* value ≤ 0.05.
